# Essential or Toxemic Dropsy

**Published:** 1901-12

**Authors:** 


### Essential or Toxemic Dropsy.

In the November 1st number of *Pediatrics,* W. P. Herrington, M. D., F. R. C. P., discusses the general anasarca observed chiefly in children which is similar to the ordinary dropsy of Bright's disease, except that there is an absence of albumin in the urine, and that the kidneys are apparently normal. He does not regard these as being instances of abnormal nephritis, but rather as cases of dropsy directly due to the action upon the capillaries of some toxic substance circulating in the blood. He believes also that many cases of fatal scarlatinal dropsy in which the kidneys are found to be inflamed are due to the specific poison of the disease rather than to kidney insufficiency, and that therefore the kidney inflammation is merely a coincident effect of the same cause.

